# HPV in exhaled breath condensate of lung cancer patients

**DOI:** 10.1038/bjc.2011.354

**Published:** 2011-09-27

**Authors:** G E Carpagnano, A Koutelou, M I Natalicchio, D Martinelli, C Ruggieri, A Di Taranto, R Antonetti, F Carpagnano, M P Foschino-Barbaro

**Affiliations:** 1 Institute of Respiratory Disease, Department of Medical and Occupational Sciences, Faculty of Medicine, University of Foggia, Foggia, Italy; 2 III Laboratory of Analysis, Department of Clinical Pathology, Riuniti Hospital Foggia, Foggia, Italy; 3 Department of Medical Sciences, Section of Hygiene, Faculty of Medicine, University of Foggia, Apulia Regional Epidemiological Observatory, Foggia, Italy; 4 Department of Thoracic Surgery, Case di Cura Riunite, La Madonnina, Bari, Italy

**Keywords:** human papilloma virus, bronchial brushing, breath condensate, lung cancer

## Abstract

**Background:**

A recent intriguing carcinogenetic hypothesis for lung cancer foresees its viral aetiology. The human papilloma virus (HPV) is the main virus actually recognised in the pathogenesis of lung cancer. The aim of this study was to investigate, for the first time to our knowledge, the presence of HPV in the exhaled breath condensate (EBC) of lung cancer patients.

**Materials and method:**

We enrolled 89 patients affected by lung cancer and 68 controls. HPV infections were investigated in their EBC, paired bronchial brushing and neoplastic lung tissue through genotyping.

**Results:**

We were able to detect HPV in the EBC, bronchial brushing and neoplastic lung tissue. We described the presence of an HPV infection in 16.4% of the subjects affected by non-small cell lung cancer, but in none of the controls. HPV 16 and 31 turned out to be the most widespread genotypes. The HPV positivity in airways as well as in the smoking habit was seen to independently increase the individual's susceptibility to developing lung cancer.

**Conclusion:**

When summing up, we demonstrated the possibility to identify an HPV infection in the EBC of lung cancer patients; further, we supported the notion that the EBC is a suitable tool to study airway colonisation. That being said, although further studies are needed to confirm our results, we retain the study of HPV in EBC to be very interesting in terms of future programmes involving lung-cancer screening.

A viral aetiology of lung tumours is a tantalising prospect since the virus can be regarded as a main oncogenic event or as an important co-factor (Brouchet *et al*, 2005). At another level, the possibility that the human papilloma virus (HPV), a small double-stranded DNA virus that belongs to the family of papillomaviridae, contributes to the pathogenesis of lung cancer is an intriguing hypothesis (Menon *et al*, 1995; De Villiers *et al*, 2004). In 1979, Syrjänen was the first to suggest that HPV was involved in lung cancer (Syrjänen, 1979). After an initial description, several other studies subsequently confirmed his findings (Tsuhako *et al*, 1998; Castillo *et al*, 2006; Ciotti *et al*, 2006; Aguayo *et al*, 2007; Nadji *et al*, 2007) until the arrival of Harald zur Hausen, who, in 2008, was conferred the Nobel Prize of physiology and Medicine for this discovery (Zur Hausen *et al*, 1974).

At present, more than 100 HPV serotypes have been identified (Cubie, 2003). The oncogenic HPV types associated with bronchopulmonary carcinoma include types 16, 18, 31, 33, whereas types 6 and 11 are non-oncogenetic (Clavel *et al*, 2000). The overall HPV prevalence ranged from 0.0 to 78.3% with large heterogeneity across geographic regions and histological tissue types (Srinivasan *et al*, 2009). The incidence of HPV in lung cancer in Europe is 17% (Klein *et al*, 2009).

Human papilloma virus infection and metaplasia in lung tissue may increase an individual's susceptibility to tumourigenesis (Chen *et al*, 2004). The key oncogenic mechanism of HPV is mediated by its ability to interfere with the cell-cycle and tumour-suppressive function of the cell through its oncoproteins E6 and E7 that inactivate p53 and Rb proteins, respectively (Werness *et al*, 1990; Kessis *et al*, 1993; Zur Hausen, 2001, 2002).

The presence of HPV in the airways of lung cancer patients has already been investigated through research of the virus in bronchial aspirates (Papadakis *et al*, 2002; Branica *et al*, 2010), fresh lung tissue (Papadopoulou *et al*, 1998; Clavel *et al*, 2000; Zafer *et al*, 2004; Coissard *et al*, 2005; Ciotti *et al*, 2006; Wang *et al*, 2008) and paraffin-embedded lung tissue (Béjui-Thivolet *et al*, 1990; Shamanin *et al*, 1994; Szabó *et al*, 1994; Hirayasu *et al*, 1996; Bohlmeyer *et al*, 1998; Tsuhako *et al*, 1998; Kaya *et al*, 2001; Syrjänen, 2002; Fei *et al*, 2006). However, confirming studies on these samples is difficult, as they are invasive and poorly accepted by patients.

An increasing interest in the study of lung cancer is currently being directed to the use of techniques that allow collecting non-invasiveness samples such as exhaled breath condensate (EBC).

Several soluble and genetic markers have already been measured in the EBC of lung cancer patients and investigated as early neoplastic markers, although none of them have been validated sufficiently for clinical application. In regards to the possibility to analyse viral or bacterial colonisation in EBC, studies on pseudomonas, burkholderia and the influenza virus were found to be unsuccessful (Vogelberg *et al*, 2003; St George *et al*, 2010).

The aim of this study was to investigate, for the first time to our knowledge, the presence of HPV in the EBC of lung cancer patients. In order to verify more effectively whether EBC reflects airways infection, we analysed the HPV in EBC, paired bronchial brushing and neoplastic lung tissue of those patients affected by lung cancer and of the controls.

## Materials and methods

### Characteristics of the patients

A total of 157 consecutive patients with a suspicion of lung cancer (44 F, 118 M, 66.9±11.6 years) that consented to the study were enrolled at the Unit of Thoracic Surgery, Casa di Cura La Madonnina, Bari, as well as at the Department of Respiratory Disease, Foggia University (Table 1). Written informed consent was obtained from all subjects upon approval of the study by the Ethics Committees of the two hospitals.

All patients were enrolled in the study before pathological diagnosis. All patients also underwent standard staging procedures consisting in a physical examination, serum chemistry analysis, brain, chest and abdomen CT scans and radionuclide bone scan.

All the patients underwent thoracotomy except for six of them, who were deemed surgically inoperable for reasons related to staging and/or inadequate cardiorespiratory functioning.

In these cases the definitive diagnosis of malignancy derived from a positive cytohistology of the samples obtained broncoscopically. Following the histological analysis carried out on specimens, 68 subjects turned out to be negative and were considered as controls. All 68 patients were operated on and the definitive diagnosis is shown in Table 1. In the remaining 89 subjects, the suspicions of lung cancer were confirmed. Squamous cell carcinoma was diagnosed in 36 subjects, whereas 37 subjects were found to be affected by adenocarcinoma and 16 by small-cell lung cancer. Overall the patients were classified as stage I in 18 cases, stage II in 15 cases, stage III in 25 cases and stage IV in 21 cases.

All subjects underwent EBC and bronchial brushing collection (the latter was carried out during bronchoscopy). Their paraffin-embedded neoplastic lung tissue, collected during lung surgery, was stored for successive analysis.

We acquired information on their smoking habit at the time of diagnosis. In all, 67 of the lung cancer patients were current smokers (43.5±11.6 packs per year), whereas 17 were ex smokers (42.9±18.6 packs per year) and 5 were non-smokers. On the other hand, in the controls group fifty-one were current smokers (40.6±10.3 packs per year), eight were ex smokers (39.9±16.5 packs per year) and nine were non-smokers. A detailed history relating to their family history of lung cancer or any other cancer was collected in a pre-tested pro-form. All the women enrolled underwent a PAP smear test and were evaluated as cytological using the Bethesda system for PAP smear interpretation along with colposcopic evaluation. We considered negative samples where koilocytosis was absent.

### Bronchial brushing collection and processing

Bronchial brushing specimens were collected in ice-cold phosphate-buffered saline solution during the fibreoptic bronchoscopy and were stored at −70 °C for subsequent analysis.

DNA extraction was performed using qiasymphony (Qiagen, Hilden, Germany), following the manufacturer's instructions.

### EBC collection and processing

The above required 1 ml of EBC in one setting from each patient at the time of diagnosis.

The EBC was collected by using a condenser, which allowed for the non-invasive collection of the non-gaseous components of the expiratory air (EcoScreen Jaeger, Wurzburg, Germany). The condensate was collected in ice at −20 °C, transferred to 1.5 ml polypropylene tubes and immediately stored at −70 °C for subsequent analysis.

DNA extraction was performed using qiasymphony (Qiagen) following the manufacturer's instructions.

### Paraffin-embedded lung tissue processing

Formalin-fixed and paraffin-embedded neoplastic lung tissue were sectioned at a thickness of 10 *μ*m and used for each patient for DNA extraction purposes.

At the beginning, one slide of lung tissue was treated with ematossilin eosin for the selection of the neoplastic lung area. Next, from the area selected neoplastic material was asported and put into two separate 1.5-ml polypropylene tubes. After that samples were deparaffinised in xylene and then rehydrated through serial dilutions of alcohol. After evaporation, the DNA was extracted and purified with a DNA Qiamp mini-kit (Qiagen) that foresaw the first step of enzymatic digestion with an ATL buffer and proteinase K overnight at 56 °C. For the second step, we added lysis buffer AL and carried out incubation at 70 °C for 10 min, whereas the further step involved purification by the addition of absolute ethanol and passage through ion-exchange column chromatography followed by washing and finally the use of elution buffer AE with DNA in a volume proportional to the starting material.

### Measurement of HPV DNA

The HPV DNA was analysed in DNA from EBC, bronchial brushing and lung tissue.

The assay for HPV genotyping was initially performed using an HPV sign (Diatech, Jesi, Italy).

The DNA was amplified by PCR reactions with one of the primers biotinylated on Rotor-Gene TM 6000 (Corbett Research, Sydney, Australia), whereas single-stranded DNA templates were prepared using the PyroMark Vacuum Prep Workstation (Biotage, Uppsala, Sweden).

The pyrosequencing analysis was performed on the PyroMarkTM Q96 ID instrument (Biotage).

**Figure 1:**
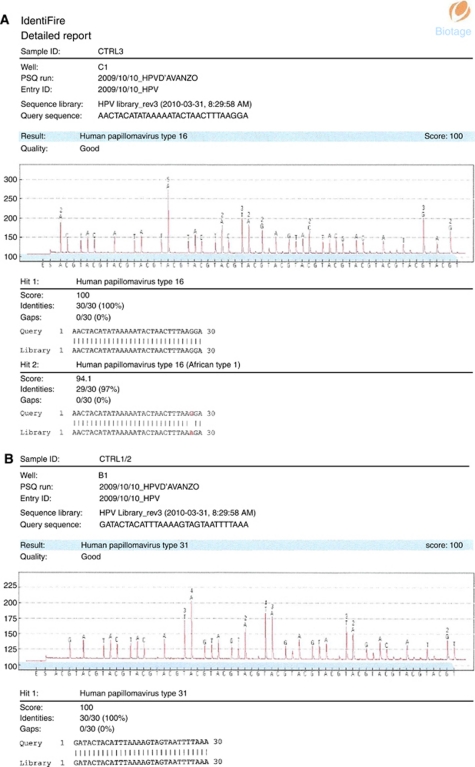
HPV 16 (**A**) and HPV 30 (**B**) pirograms.

**Figure 2:**
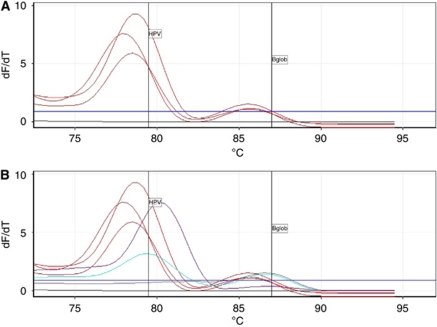
Melt curves (**A** and **B**).

Pyrosequencing is a DNA-sequencing technique to determine nucleic acid sequences. This method is a sequencing-by-synthesis technique that employs a series of enzymatic reactions to accurately detect short nucleic acid sequences during DNA synthesis. In principle, when a complementary nucleotide is incorporated by DNA polymerase into an already-primed DNA template (whereby the sequencing primer is hybridised to target the DNA), an inorganic pyrophosphate (PPi) molecule is released. The released PPi is converted to ATP by ATP sulphurylase using adenosine phosphosulphate as substrate. This reaction provides energy for the conversion of luciferase to oxidase luciferin, and consequently the light produces a peak in the pyrogram (c.f. electropherogram in Sanger sequencing). Each signal peak is proportional to the number of nucleotides incorporated (e.g., a triple dTTP nucleotide incorporation generates a triple higher peak). Apyrase is a nucleotide-degrading enzyme, which continuously degrades the ATP and non-incorporated dNTPs in the reaction mixture. There is a certain time interval between each nucleotide dispensation to allow for complete degradation. For this reason, one nucleotide addition is performed at a time. During the sequencing-by-synthesis process, the primed-DNA strand is extended by complementary nucleotides, and the DNA-sequence signals are displayed by the signal peaks in a pyrogram on a computer monitor. Base callings are performed with integrated software with features for related SQA and sequencing analysis. HPV 16 and HPV 30 pirograms and relative melt curves are reported in Figures 1 and 2.

The INFINITI HPV-QUAD assay was successively used to confirm the HPV results.

The AutoGenomics HPV-QUAD assay targets the *E1* gene of the HPV genome. Multiplex PCR for five individual HPV types (16, 18, 31, 33 and 45), five combinations of HPV types (35/68, 39/56, 58/52, 59/51 and 6/11) and a *β*-globin internal control were performed on extracted DNA using Platinum TaqDNA Polymerase (Invitrogen, Carlsbad, CA, USA) and an amplification mix was provided with the INFINITI HPV-QUAD Assay (AutoGenomics, Carlsbad, CA) as previously reported (Erali *et al*, 2009).

### Statistical analysis

In order to assess the association between categorical variables such as sex, smoking habit or HPV positivity and positivity to lung cancer, or between histological subtypes or TNM stage and HPV positivity, the *χ*^2^-test or (Fisher's exact test, when it was necessary) and odds ratio (OR) with relative confidence intervals (95% CI) were calculated. The *t*-Student's test for independent samples was used to assess the differences in continuous variables (age, number of packs/years smoked) between lung cancer patients and controls.

An exact logistic regression model was performed in order to state the possible confusing effect among the variables, in the comparison between the NSCLC and the healthy controls. A *P*-value of <0.05 was considered statistically significant.

Sensibilities, specificity, positive and negative predictive values with relative confidence intervals of 95% were calculated for cytology, as well as for cytology in association with the HPV test (in brushing or EBC). Statistical analysis was carried out using STATA 10 MP for the MAC OS X software package (TStat S.r.l., Sulmona, Italy). The data were analysed by the Department of Medical Sciences, Section of Hygiene, University of Foggia.

## Results

The number of lung cancer cases and healthy subjects enrolled in the present study was 89 and 68, respectively. Demographic and clinical data of study subjects are summarised in Table 1.

The male sex was associated as having NSCLC (OR=3.3, 95% CI=1.84–7.8; *χ*^2^=9.46, *P*=0.0021).

For the first time we were able to detect an HPV infection in the EBC. In only one EBC sample we observed a low concentration of viral DNA (measured spectrophotometrically and resulting as not being dosable) that did not allow for the evaluation of HPV positivity.

Eleven NSCLC patients (15.1%) turned out to be HPV positive on the EBC. Of these six were positive to HPV-16 and three to HPV-31 (genotype at high risk for cancer), one to HPV-30 and one to HPV-39. Comparing the NSCLC-positive patients with the controls who were not affected by tumours (the SCLC have been excluded from the analysis) it was shown that being HPV positive to the EBC increases the probability of being affected by NSCLC (Fisher's exact *P*=0.002, degree of freedom=1).

A total of 12 NSCLC patients (16.4%) turned out to be HPV positive on the bronchial brushing and lung tissue. Of these, seven were positive to HPV-16 and three to HPV-31 (genotype at high risk for cancer), one to HPV-30 and one to HPV-39. Comparing the NSCLC-positive patients with the controls who were not affected by tumours (the SCLC have been excluded from the analysis) it was shown that being HPV positive to the bronchial brushing and lung tissue increases the probability of being affected by NSCLC (Fisher's exact *P*=0.0011, degree of freedom=1).

Paired samples analysed (EBC, bronchial brushing and lung tissue) showed, except in one case, an overlap of the HPV positivity spectrum.

All controls turned out to be negative to HPV both in the EBC and in the paired brushing and lung tissue.

The HPV results were confirmed in all the samples of those subjects analysed with the INFINITI HPV-QUAD assay.

Comparing the NSCLCs to the healthy controls (SCLCs have been excluded from the analysis) using sex as a criterion, being smokers/ex smokers, being HPV positive on the brushing and HPV positive on the EBC as variables in logistic regression models, either being smokers/ex smokers or HPV positive (in EBC, bronchial brushing or lung tissue) will increase the risk of having an NSCLC (Tables 2, 3 and 4).

Higher percentages of HPV positivity in the EBC, bronchial brushing or lung tissue were observed in adenocarcinoma than in squamous cell carcinoma, although the difference did not reach a statistical significance (Table 5 – Fisher's test, *P*>0.05). On the other hand, none of the SCLC patients showed HPV positivity in the EBC, brushing or lung tissue. HPV-positive patients were those with TNM stage>2 (Fisher's test, *P*<0.05). Neither sex, age nor smoking habit were found to be associated with a more advanced stage of lung cancer or to HPV positivity. The association of cytology with the HPV test (in the EBC, brushing or lung tissue) slightly increases the sensibility of the test (Table 6).

## Discussion

The main result in this study was the presence of HPV positivity in the EBC, in addition to the paired bronchial brushing and neoplastic lung tissue of lung cancer patients compared with the complete HPV negativity in controls. For the first time in this study we were able to detect HPV infection in the EBC. The positivity to HPV both in the EBC and in the bronchial brushing and in the lung tissue as well as the smoking habit were found to be an independent factor in increasing the probability of causing the NSCLC. Furthermore, the association of the positivity of the cytology and the HPV test (in EBC, brushing or lung tissue) slightly increases the sensibility of malignant diagnosis.

The great interest in the viral aetiology of lung cancer recently produced several studies on blood-borne HPV in the other local samples of lung cancer patients (Béjui-Thivolet *et al*, 1990; Shamanin *et al*, 1994; Szabó *et al*, 1994; Hirayasu *et al*, 1996; Bohlmeyer *et al*, 1998; Tsuhako *et al*, 1998; Kaya *et al*, 2001; Syrjänen, 2002; Fei *et al*, 2006). Although the research of other viruses or bacteria in the EBC was previously reported as unsuccessful (Vogelberg *et al*, 2003; St George *et al*, 2010), we set out to investigate this sample for HPV in lung cancer patients or subjects at risk as smokers. The complete non-invasiveness of the EBC makes it a more tempting option as compared with other samples, especially in the field of screening subjects at risk.

In disaccordance with other studies showing that EBC is not a tool for detection of pseudomonas, burkholderia and influenza viruses, we were able to detect HPV in the EBC of NSCLC patients that were also found to be HPV positive in paired bronchial brushing and lung tissue (Vogelberg *et al*, 2003; St George *et al*, 2010). For the first time the demonstration of the presence of the HPV positivity in the EBC, paired bronchial brushing and lung tissue led us to suppose that the EBC contains identical information on airway colonisation as previously demonstrated for somatic DNA alterations specific to lung cancer (Carpagnano *et al*, 2008). Our supposition is further supported by the confirmation of our results, which were obtained using the INFINITI HPV-QUAD assay, which allows us to exclude possible false positives due to the high sensitivity of PCR.

Differences with other studies on respiratory viruses and bacteria investigation in the EBC with molecular methods could simply be due to the different pathogens that we investigated as well as differences in the nucleic acid-based techniques used (Vogelberg *et al*, 2003; St George *et al*, 2010).

The findings of the involvement of HPV infection in lung cancer are often controversial. Several authors reported that HPV has no role to play in lung cancerogenesis (Shamanin *et al*, 1994; Gorgoulis *et al*, 1999; Matakidou *et al*, 2003), whereas others observed from a low (Béjui-Thivolet *et al*, 1990; Bohlmeyer *et al*, 1998; Clavel *et al*, 2000; Kaya *et al*, 2001; Syrjänen, 2002) to moderate or to a very high frequency (Yousem *et al*, 1992; Li *et al*, 1995; Hirayasu *et al*, 1996; Tsuhako *et al*, 1998; Miyagi *et al*, 2001; Papadakis *et al*, 2002). We reported a positivity of HPV in 16% of bronchial brushing cases, paired lung tissue and the EBC of lung cancer patients, which is not only in line with previous data but supports the hypothesis of a causative link between HPV and lung cancer. Differences in HPV percentages are coherent with differences in studies regarding cancer types (NSCLC, SCLC), material (frozen or fresh lung biopsy, paraffin-embedded tissue, BAL etc), methodology (PCR, southern blot technique, *in-situ* hybridisation), inadequate choice of primers and geographic area analysed. The complete negativity reported in controls is the proof of the specificity of HPV analysis in the bronchial brushing, lung tissue and the EBC of neoplastic patients.

In consideration of the fact that HPV is the second most important cause of lung cancer after cigarette smoke, in order to better understand the co-responsibility of both of these aetiological factors we analysed the HPV infection in relation to the smoking habit and did not report any correlation. Further confirmation of our suggestion is supplied by the result of the logistic regression model applied to variables substantiating that the HPV infection is an independent factor in causing lung cancer.

We reported higher percentages of HPV positivity in lung cancer smokers than in ex smokers or non-smokers. These data found a rationale in the fact that the respiratory epithelium of smokers shows micro-abrasions with squamo-columnar junctions that are considered to be the prerequisite for the spread of HPV (Kashima *et al*, 1993).

Similar percentages of HPV positivity were reported in both females and males. The sex of patients was not found to influence HPV positivity. These findings are in contrast with those of Cheng *et al* (2004), who previously described a prevalence of HPV6 in the lung tumours of male patients, indicating that there could be HPV routes for different genders. Differences in number population and in ethnicity could explain differences between our studies, which nevertheless merit to be better investigated in a larger population.

In accordance with previous studies we reported HPV positivity both in adenocarcinoma (An *et al*, 2005) and in squamous cell carcinoma (Klein *et al*, 2009). However, despite the higher percentages of HPV positivity in brushing and EBC observed in adenocarcinoma with respect to those in squamous cell carcinoma, they did not reach statistical significance.

According to previous studies (Béjui-Thivolet *et al*, 1990; Chen *et al*, 2004; Zafer *et al*, 2004; Ciotti *et al*, 2006; Klein *et al*, 2009; Rezazadeh *et al*, 2009) we found that the genotypes most prevalent in our population were numbers 16 and 31, which are also the most oncogenic. However, we also reported one case of HPV 30 and one of HPV 39.

A further finding of this study was the higher percentage of HPV positivity in patients with a more advanced stage of lung cancer. Previously Weichert *et al* (2009) had described a high presence of HPV in patients with advanced cancer and supposed that this virus could suggest a metastatic disease, which would seem to support the notion that HPV typing, a method routinely used in cervical biopsies for some years now, could be a useful diagnostic tool for discriminating primary from metastatic carcinoma of the lung (Weichert *et al*, 2009).

The major diffusion of HPV in advanced lung cancer is conceivable as the lung cancer cells (originally HPV negative) are more prone to integrate HPV into the tumour genome than normal cells (Klein *et al*, 2009).

HPV is generally transmitted via direct mucosa contact (Stanley *et al*, 2007). In the lung it is not possible and there might be transmission via the air stream, carrying infected cell complexes or particles to the periphery of the lung. Chiou *et al* (2003) reported the detection of HPV 16/18 DNA in the blood, opening our insight into some transmission routes of HPV that are potentially distinct from direct mucosa contact. However, although all the women in our study investigated for eventual HPV positivity to the PAP test resulted negative, a limitation of this study was not exploring the HPV in the blood, which would have permitted us to discuss the hypothesis of systemic transmission. A further limitation of this study was not testing the HPV in partners to exclude the other largely recognised rue of transmission, that is, the sexual one (Chen *et al*, 2004; Georgieva *et al*, 2009).

Finally, we observed that associating the HPV test in the EBC, brushing or lung tissue slightly increases the sensibility of the cytology. This finding justifies us in encouraging the undertaking of further studies on this field, as they could offer new, more sensitive screening instruments for lung cancer, a disease still characterised by poor results in terms of early diagnosis.

In conclusion, we can be said to have demonstrated that HPV infection is detectable in the EBC of lung cancer patients and that the EBC could be considered a suitable tool for studying the viral colonisation of airways. The EBC and paired bronchial brushing and lung tissue showed an overlap of the HPV-positivity spectrum that was found to be independent from cigarette smoking in increasing the individual's susceptibility to develop lung cancer. Although it could be useful to confirm our results on a larger population, we retain them very interesting in prospective of what they could represent in terms of the early screening of lung cancer patients and possible program of cancer prevention, such as those already commonly used for cervical cancer.

In consideration of the importance of characterising the role of HPV in lung cancer, we are planning to analyse the HPV physical status (episomal/integrated) in the EBC, bronchial brushing and lung tissue of NSCLC patients in a future study.

## Figures and Tables

**Table 1:** Demographic and clinical data of subjects enrolled

	**Lung cancer patients**	**Healthy subjects**
*N*	89	68
Sex (M/F)	72/17	41/27
Age (years)	68.2±11.3	66.8±4.8
FEV1 (%)	92.2±4.1	91.3±3.4
FVC (%)	97.2±3.1	96.1±2.9
		
*Histological type*		
Squamous cell carcinoma	36	—
Adenocarcinoma	37	—
Small cell lung cancer	16	—
Bronchial pneumonia	—	32
Bronchogenic cyst	—	2
Hamartomas	—	6
Mycetomas	—	7
Pulmonary abscess	—	7
Pulmonary tuberculoma	—	6
Endothoracic neurinoma	—	3
Klemperer's tumour	—	2
Typical carcinoid	—	3
	—	
		
*TNM stage of lung cancer*		
I	18	—
II	15	—
III	28	—
IV	21	—
		
*Smoking habits*		
Current smokers	67	51
Ex-smokers	17	8
Non-smokers	5	9
		
*PAP test*		
Positive	0	0
Negative	24	17

Abbreviations: F=female; FEV1=forced expiratory volume in 1 second; FVC=forced vital capacity; M=male; PAP=papanicolaou; TNM=tumour nodes metastasis.

**Table 2:** Number of NSCLC subjects and controls by sex, smoking habits and HPV+ (in the brushing and lung tissue or in the EBC), ORs (95% CIs) and *P*-values

	**Controls**	**NSCLC**	**Total**	***P***	**OR (95% CI)**
*Sex*					
F	27	12	39	0.002	3.34 (1.4–8.1)
M	41	61	102		
Total	68	73	141		
					
*Smokers/ex-smokers* a					
N	28	12	40	0.001	3.6 (1.5–8.5)
Y	40	61	101		
Total	68	73	141		
					
*HPV+ in the brushing and lung tissue*					
N	68	61	129	0.0005	—
Y	0	12	12		
Total	68	73	141		
					
*HPV+ in the EBC*					
N	68	62	130	0.0009	—
Y	0	11	11		
Total	68	73	141		

Abbreviations: CI=confidence interval; EBC=exhaled breath condensate; F=female; HPV=human papilloma virus; M=male; N=no; NSCLC=non-small cell lung cancer; OR=odds ratio; Y=yes.

a Average number of cigarette packs per year: 41.8±18.5.

**Table 3:** Number of HPV+/− (in the brushing and lung tissue or in the EBC) subjects by sex and smoking habits, ORs (95% CIs) and *P*-values

	**N**	**Y**	**Total**	***P* (Fisher)**	**OR (95% CI)**
*HPV+ in the brushing and lung tissue*					
Sex					
F	37	2	39	>0.05	2 (0.4–19.7)
M	92	10	102		
Total	129	12	141		
					
*Smokers/ex-smokers* a					
N	33	7	40	0.04	0.3 (0.06–0.97)
Y	96	5	101		
Total	129	12	141		
					
*HPV+ in the EBC*					
Sex					
F	38	1	39	>0.05	4.1 (0.5–183.9)
M	92	10	102		
Total	130	11	141		
					
*Smokers/ex-smokers* a					
N	34	6	40	0.05	0.3 (0.07–1.25)
Y	96	5	101		
Total	130	11	141		

Abbreviations: CI=confidence interval; EBC=exhaled breath condensate; F=female; HPV=human papilloma virus; M=male; N=no; OR=odds ratio; Y=yes.

a Average number of cigarette packs per year: 41.8±18.5.

**Table 4:** (a) Exact logistic regression analysis between NSCLCs/controls (dependent variable) and sex, smoking habits and HPV+ in the brushing and lung tissue (independent variables), (b) logistic regression analysis between NSCLCs/controls (dependent variable) and sex, smoking habits and HPV+ in the EBC (independent variables)

	**OR**	***P***	**95% CI**
*(a)*			
Sex (M)	1.9	0.182	0.74–5.01
Smokers/ex-smokers^a^	6.89	0.0001	2.3–25.25
HPV+ in the brushing and lung tissue	42.8	<0.0001	From 6 to +∞
			
*(b)*			
Sex (M)	1.8	0.197	0.7–4.5
Smokers/ex-smokers^a^	5.8	0.0002	2.1–19.2
HPV+ in the EBC	30.7	0.0001	From 4.3 to +∞

Abbreviations: CI=confidence interval; EBC=exhaled breath condensate; HPV=human papilloma virus; M=male; NSCLC=non-small cell lung cancer; OR=odds ratio.

a Average number of cigarette packs per year: 41.8±18.5.

**Table 5:** NSCLCs by histotype and HPV+ in the brushing and lung tissue or HPV+ in the EBC

	**NSCLCs**						
	**Squamous**		**Adenocarcinoma**				
	***N***	**%**	***N***	**%**	***N***	***P* (Fisher)**	**OR (95% CI)**
*HPV+ in the brushing and lung tissue*							
No	33	89.2%	28	77.8%	61	>0.05	0.4 (0.08–1.8)
Yes	4	10.8%	8	22.2%	12		
Total	37		36		73		
							
*HPV+ in the EBC*							
No	33	89.2%	29	80.6%	62	>0.05	0.5 (0.1–2.2)
Yes	4	10.8%	7	19.4%	11		
Total	37		36		73		

Abbreviations: CI=confidence interval; EBC=exhaled breath condensate; HPV=human papilloma virus; M=male; NSCLC=non-small cell lung cancer; OR=odds ratio.

**Table 6:** Sensitivity and specificity of tests

	**Sensitivity (95% CI), %**
Cytology^a^+	66 (55–77)
Cytology^a^+ and HPV+ in the brushing	73 (62–83)
Cytology^a^+ and HPV+ in the lung tissue	73 (62–83)
Cytology^a^+ and HPV+ in the EBC	73 (62–83)

a Brushing cytology.
